# A Comparative Study between Microwave Hydrodiffusion and Gravity (MHG) and Ultrasound-Assisted Extraction (UAE): Chemical and Biological Characterization of Polyphenol-Enriched Extracts from Aglianico Grape Pomace

**DOI:** 10.3390/foods12142678

**Published:** 2023-07-11

**Authors:** Giuseppina Crescente, Giovanni Cascone, Antonio Petruzziello, Aziz Bouymajane, Maria Grazia Volpe, Gian Luigi Russo, Stefania Moccia

**Affiliations:** 1 National Research Council, Institute of Food Sciences, 83100 Avellino, Italy; giuseppina.crescente@isa.cnr.it (G.C.); giovanni.cascone@isa.cnr.it (G.C.); antonio.petruzziello@isa.cnr.it (A.P.); mgvolpe@isa.cnr.it (M.G.V.); glrusso@isa.cnr.it (G.L.R.); 2 National Research Council, Research Institute on Terrestrial Ecosystems, 80131 Napoli, Italy; azizbouymajane.01@gmail.com

**Keywords:** grape pomace, antioxidant compounds, microwave hydrodiffusion and gravity extraction, ultrasound-assisted extraction, polyphenols, antiproliferative activity

## Abstract

The wine industry produces large amounts of grape pomace (GP), a waste that needs to be disposed of properly. Bioactive compounds with high added value can be recovered from GP as an interesting strategy to reduce the environmental impact. Here, two different technologies were employed to recover polyphenol compounds from GP: microwave hydrodiffusion and gravity (MHG) and ultrasound-assisted extraction (UAE). The further purification of UAE and MHG extracts was carried out through solid-phase extraction (SPE) to obtain three fractions, F1, F2 and F3. ATR-FTIR analysis confirmed the presence of sugar and polysaccharide components in F1, as well as non-anthocyanin and anthocyanin compounds in F2 and F3, respectively. Also, the chemical profile was determined by HPLC-UV-DAD, identifying the presence of catechin in F2, and malvidin-3-*O*-glucoside chloride and cyanidin chloride derivative as the main anthocyanin compounds in F3. The fractions and their parental extracts were characterized for total phenolic content (TPC) and scavenger activity by *in vitro* assays. We found that F2-MHG and F3-MHG contained phenol contents 6.5 and 8.5 times higher than those of the parental non-fractionated extracts. Finally, F3-MHG (100 μg/mL, *w*/*v*) was shown to reduce the proliferation of HT-29 cells.

## 1. Introduction

Food and agriculture chains produce huge amounts of waste, which are often improperly disposed of. From a circular economy perspective, “green chemistry” with zero waste is attracting great interest in the efficient reuse of waste materials. In most cases, these residues contain bioactive compounds, specifically polyphenols, that are widely applicable [1,2,3]. 

To recover these compounds from agro-industrial wastes, different extraction techniques can be used, existing processes can be improved and optimized, and new methods and procedures can be implemented, including the use of alternative solvents [2].

Green chemistry-based extraction technologies are preferred over conventional ones, which present several critical issues (extraction time, solvent consumption, etc.) and usually break eco-extraction principles [4,5]. The use of novel techniques such as microwave hydrodiffusion and gravity (MHG) or ultrasound-assisted extraction (UAE) to recover high-added value components from agricultural by-products represents a valuable alternative. 

MHG is one the most innovative applications of Solvent-Free Microwave Extraction (SFME); it is an efficient, quick, and environmentally friendly method of extracting phenolic compounds without using solvents [6]. It was originally developed to extract essential oils, but it is currently being explored for the extraction of polyphenols from raw plant materials. Due to its eco-friendliness and economic benefits, it has gained considerable attention for use in recovering bioactive molecules from agricultural wastes or by-products for developing innovative products [7,8]. Because of the short length of the procedure and the excellent temperature control, microwave radiation does not cause degradation of the thermolabile compounds [9]. 

An alternative approach is UAE, a technique widely used to extract polyphenols that involves less time while enhancing extraction yield and extract quality. Compared to the MHG, in the case of UAE, solvents are necessary to extract bioactive compounds effectively [10].

One of the most significant agro-food chains that significantly impact global economies is the wine chain, which involves recovering and valuing waste materials and by-products. There has been a growing interest in utilizing the by-products of the wine industry in recent years. The winemaking process, although considered environmentally friendly, generates many residues, consisting mainly of vine stems, grape pomace (GP), and wine lees [11,12]. GP represents approximately 20% to 25% of the weight of the grapes used, and it is generated after fermentation using red grapes, while when using white ones, it is generated before [13,14]. It contains a high level of bioactive molecules; in fact, about 70% of the grapes’ phenolic compounds remain in the pomace after the winemaking process has been completed [12,15]. Several factors play a role in the chemical composition of GP, including the grape variety, agronomic conditions, and winemaking practices [16]. However, the principal phenolic components of GP are phenolic acid (i.e., hydroxybenzoic and hydroxycinnamic acids), flavonoids (flavanols, flavonols, anthocyanins), stilbenes (resveratrol and its derivatives) and tannins [17]. Since these compounds have been proven to be antioxidant, anti-inflammatory, and anti-ageing compounds, GP-derived products can be appealing for incorporation into the human diet [13]. 

The biological efficacy of GP extract has been reported on human colorectal cancer cell lines, highlighting a pro-apoptotic mechanism [18,19,20]. To our knowledge, no data are available on the biological capacity of polyphenolic fractions (mainly anthocyanins) extracted from GP through MHG, especially from the Aglianico cultivar. 

Following the dictates of green extraction, we here compare the chemical and biological profiles of two different techniques used to recover polyphenols from the GP of the Aglianico cultivar. Firstly, GP was extracted using the MHG technique without solvent; in parallel, UAE with a hydroalcoholic solution was carried out. As a result, the differences between the two extractive approaches are mirrored in the significantly different behaviours of the two parental extracts. In this context, fractionation strategies have been further employed. The chemical composition was unravelled by Attenuated Total Reflectance–Fourier Transform Infrared Spectroscopy (ATR-FTIR) and high-performance liquid chromatography–ultraviolet–diode array detection (HPLC-UV-DAD) analysis, and the extracts and fractions obtained were further evaluated for their phenolic contents and scavenger activities. Considering that bioactivity assessment is closely related to chemical composition, the differences in biological efficacy between the two extraction methods can be attributed to the differences in their polyphenol profile. We reported that the GP polyphenol fraction produced by MHG, including the anthocyanin-enriched fraction, significantly reduced malignant cell growth, strongly suggesting its antiproliferative potential. For this reason, MHG represents an interesting approach for the preparation of non-degraded, polyphenol-rich GP extracts, which could find applications in several fields. Here, we provide for the first time a chemical–biological validation of polyphenol fractions from GP obtained by MHG, which may complement or serve as a valid alternative to the UAE technique still widely used for polyphenolic compound extraction. 

## 2. Materials and Methods

### 2.1. Grape Pomace (GP) Sampling and Reagents

The GP (*Vitis vinifera* L., Aglianico cultivar) was supplied by an experimental winery in Taurasi (Avellino, Italy) in October 2022; it was frozen on the same day as the wine was produced. The grapes of the Aglianico cultivar are the main variety used in the production of “Taurasi” DOCG (Appellation of Controlled and Guaranteed Origin) wines. All chemicals were of analytical grade, and they were acquired from Merck (Milan, Italy).

### 2.2. Microwave Hydrodiffusion and Gravity Extraction (MHG)

The MHG extraction was performed using an advanced microwave extraction system ETHOS X (Milestone, Bergamo, Italy) in the “Flavors set-up” configuration. Briefly, 400 g of frozen GP was homogenized with 320 mL of H_2_O_d_ (80% *v*/*w* of GP), placed in the extraction vessel, and subjected to 800 W (Power Density = 2 W/g) under atmospheric pressure. The temperature was monitored using an internal infrared sensor (coupled to the MHG), reaching 80 °C at the end of the extraction. Two extracts were produced, the first of which was coloured, and the second one was colourless. Only the first extract was collected as in [4]; therefore, the procedure was completed when the extract became colourless (about 10 min). The yield was calculated based on the volume of coloured extract and expressed in % (*v*/*w*) of extract over the GP mass.

### 2.3. Ultrasound-Assisted Extraction (UAE)

UAE was performed using a Branson Ultrasonics^TM^ CPX1800H-E (Danbury, CT, USA). Briefly, 10 g of frozen GP were homogenized in MeOH/H_2_O (1:1, *v*/*v*; Ultra-Turrax T25 blender; 20,000 rpm; 1 min) and underwent UAE for 30 min. The raw material/solvent ratio was 1:5 (g raw material: mL solvent). The ultrasound bath temperature was set at 20 °C. Following centrifugation (Neya 16R; 8000 rpm for 20 min at 4 °C), the supernatant was stored. 

### 2.4. Solid-Phase Extraction (SPE) 

**Figure 1:**
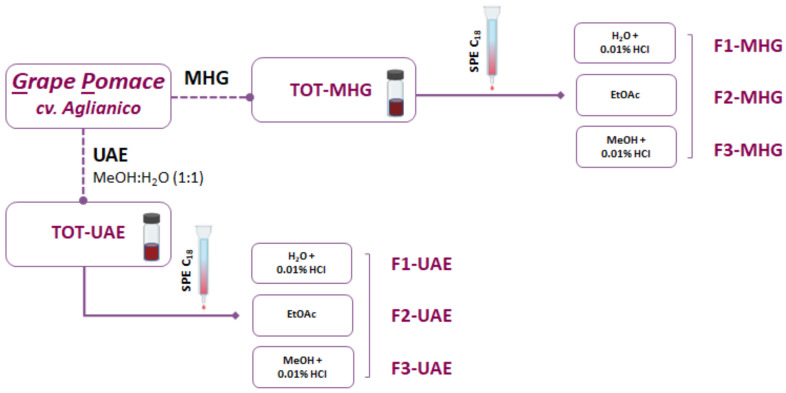
Flow diagram of extraction and fractionation of non-anthocyanins and anthocyanins in GP cv. Aglianico (UAE = ultrasound-assisted extraction; MHG = microwave hydrodiffusion and gravity; SPE = solid-phase extraction).

Both extraction methods yielded different extracts (TOT-UAE and TOT-MHG, respectively) that were further fractionated using a SePak C-18 cartridge (Waters, Milford, MA, USA) according to [21] with slight modifications. The columns were previously activated by sequentially passing 5 mL of ethyl acetate, acidified methanol (0.01% *v*/*v* HCl), and 2 mL of acidified water (0.01% *v*/*v* HCl). The extracts TOT-UAE and TOT-MHG were passed through the cartridges and eluted with 2 mL acidified water (0.01% HCl, *v*/*v*) to obtain Fractions 1 (F1-UAE and F1-MHG); after that, the cartridges were rinsed with 5 mL of ethyl acetate, to elute phenolic compounds other than anthocyanins (Fraction 2, F2-UAE and F2-MHG). Acidic methanol was used to elute the adsorbed anthocyanins from the SePak cartridges (Fraction 3, F3-UAE and F3-MHG). Finally, the solvent of all fractions was evaporated, and the samples were stored at −20 °C for further analysis. The experimental procedure is illustrated in Figure 1.

### 2.5. Characterization of UAE and MHG Fractions 

#### 2.5.1. ATR-FTIR Analysis

The ATR-FTIR analysis was performed on TOT-UAE, F1-UAE, F2-UAE, F3-UAE, TOT-MHG, F1-MHG, F2-MHG, and F3-MHG using a Spectrum 400 spectrophotometer (PerkinElmer, Waltham, MA, USA), equipped with a deuterated tri-glycine sulfate (DTGS) detector, as described in [22], with minor modifications. Briefly, 1 µL of the liquid sample was placed directly on the surface of the germanium crystal and it was allowed to dry for 10 min at room temperature (about 20 °C), or until the recorded spectrum stabilized. After each spectrum collection, the crystal surface was cleaned with 0.1% (*w*/*v*) Alconox solution (Alconox Inc., New York, NY, USA). The ATR-FTIR spectra were recorded at resolutions of 8 cm^−1^ with 32 scans in the mid-IR region (4000–650 cm^−1^). Analyses have been conducted on triplicate samples for each extract.

#### 2.5.2. Acid Hydrolysis of Anthocyanins

The anthocyanin fractions (F3-UAE and F3-MHG) obtained from SPE were subjected to acid hydrolysis according to [23]. In the first step, 1 mL of the purified anthocyanin solution was diluted with 5 mL of 2 N HCl and was hydrolyzed at 100 °C for 45 min. Afterwards, a bath of ice was used to cool the solution. The hydrolyzed product was further purified using a SePak C-18 cartridge (Waters) as described in Section 2.4.

#### 2.5.3. HPLC-UV-DAD Analysis

The HPLC-UV-DAD analysis was performed on F2-UAE, F3-UAE, F2-MHG, and F3-MHG using a 1260 Infinity II LC System (Agilent, Santa Clara, CA, USA) equipped with an Agilent G7111A quaternary pump and a WR G7115A diode array detector. Poroshell 120 EC-C18 was used for the separation (150 × 4.6 mm i.d., 4.0 μm particle size, Agilent, Santa Clara, CA, USA) with the column at 30 °C, using water (mobile phase A) and acetonitrile (mobile phase B), both with 0.02% trifluoroacetic acid. The elution condition involved the following linear gradient: 0–2 min, 0→0% B; 2–14 min, 0→18% B; 14–24 min, 18→25% B; 24–60 min, 25→58% B; 60–65 min, 58→100% B. Phase B then returned to the initial conditions and was re-equilibrated for 1 min. The total analysis time was 66 min, the flow rate was 0.350 mL/min, and the injection volume was 10 μL. Three wavelengths (280, 320, and 520 nm) were chosen for UV detection. Retention times and spectral data were compared to standards to perform identification. 

#### 2.5.4. Total Phenol Content (TPC) 

TPC was determined by the Folin–Ciocalteu method, as reported by [24]. Aliquots of parental extracts (0.25 and 0.5 mg) and fractions derived from them (0.125 and 0.25 mg) were mixed with 0.25 mL of Folin–Ciocalteu reagent and 2.25 mL of Na_2_CO_3_ (7.5%, *w*/*v*). The reaction mixture was incubated at 37 °C for 90 min. Subsequently, absorbance was measured at 765 nm (Synergy HT microplate reader, BioTek, Milan, Italy). Data are expressed as mg of gallic acid equivalent (GAE)/g of extract.

#### 2.5.5. DPPH and ABTS Radical Scavenging Activity 

Increasing doses of parental extracts (2.5, 10, 25.0, 50.0, and 100.0 μg/mL) and fractions derived from them (0.19, 0.39, 0.78, 1.56, 3.125, 6.25, 12.5, and 25 μg/mL) were estimated towards ABTS [2,2′-azinobis-(3-ethylbenzothiazolin-6-sulfonic acid)] radical cation and 2,2-diphenyl-1-picrylhydrazyl (DPPH) radical [13,25]. 

DPPH^●^ methanol solution (0.1 mM) was added to the samples; after stirring for 20 min, spectrophotometer (Synergy HT BioTek) measurements were performed at 517 nm compared to a blank [22]. 

The radical cation ABTS was produced from the reaction of 2,2′-azinobis-(3-ethylbenzothiazolin-6-sulfonic acid; 7 mM) with potassium persulfate (K_2_S_2_O_8_; 2.45 mM) for 12 h in the dark, and subsequently combined with Phosphate-Buffered Saline (PBS) until the absorbance at 734 nm reached 0.7. After 6 min, the absorbance was measured using a spectrophotometer (Synergy HT BioTek) in reference to a blank [25]. Trolox was used as a positive standard in both antiradical assays. The results are presented as percentages by considering the reduction in initial radical adsorption in the tested samples.

### 2.6. Cell Viability 

The HT-29 cell line, derived from a human colorectal adenocarcinoma, was used as a cellular model [26]. HT-29 cells were cultured in Dulbecco’s modified Eagle’s medium (DMEM) fortified with 10% fetal bovine serum (FBS; Lonza, Belgium), 1% L-glutamine, 1% penicillin and 1% streptomycin (Life Technologies, Carlsbad, CA, USA) at 37 °C, in a 5% CO_2_ humidified atmosphere. For viability assessment, the cells were plated in 96-multiwell plates at a density of 1 × 10^4^/mL and allowed to be adhered to for 24 h. The viability of cells was measured using crystal violet dye for 72 h of treatment with F1, F2, and F3 of UAE and MHG (100 μg/mL, *w*/*v*) [27,28]. Control cells (CTRL) were treated with dimethyl sulfoxide (DMSO), the solvent in which the fractions were dissolved. Briefly, HT-29 cells were washed with PBS and fixed in 10% formalin for 15 min at room temperature. After 15 min, the cells were stained with 0.02% crystal violet (*w*/*v*) for 30 min. At the end of incubation, the cells were washed and photographed in a bright field (BF) (magnification 200× using an inverted microscope Axiovert 200 Zeiss, Jena, Germany). For the quantification, the cells were solubilized with 10% acetic acid and the absorbance at 590 nm was measured spectrophotometrically. Data are reported as a percentage (%) of cell viability (CV) with respect to CTRL. Cell viability can also be determined using CyQuant DNA-binding dye [29]. Briefly, CyQuant nuclear dye and the background suppressor were added as a mixture to cells and incubated for 1 h at 37 °C. The spectrophotometer was used to measure the fluorescence at 485 and 530 nm (Synergy HT BioTek) to calculate the percentage (%) of fluorescence with respect to CTRL. 

### 2.7. Statistical Analysis

The analysis of all experiments was carried out using one-way ANOVA followed by Bonferroni’s multiple comparisons test. The GraphPad Prism version 9.5.1 for Windows 11, GraphPad Software, San Diego, CA, USA, www.graphpad.com, was used (accessed on 17 May 2023). *p*-values less than 0.05 were considered statistically significant. Data collected from duplicate, triplicate, or quadruplicate experiments have been reported as mean + standard deviation (SD). Statistical significance is indicated in the figure legends with * *p* < 0.05, ** *p* < 0.01, *** *p* < 0.001.

## 3. Results and Discussion

### 3.1. Optimization of Operative Parameters of the MHG and UAE Extraction

A preliminary investigation was conducted to determine the best extraction conditions of the two techniques in terms of time, yield, and compounds preservation. 

For the MHG extraction, the optimal power density was chosen; as reported in previous studies, it is inefficient to extract phenolic compounds with a power below 1.5 W/g, and it is harmful to do so with a power above 2.5 W/g [30]. For this reason, in atmospheric pressure conditions, a power density of 2 W/g was adopted, and in less than 10 min, the whole-coloured fraction of the GP was extracted.

Furthermore, the efficiency of MHG extraction is dependent on several factors, including the moisture of the plant material, the sample integrity, the temperature, and the length of the extraction [30,31]. It is important to humidify the plant sample before extraction in case little moisture is present [32]. 

As part of the present study, various percentages of water were tested in relation to the amount of processed GP, ranging from 25% to 80%. It was observed that below 25%, the microwave extraction process was not very effective due to the low water concentration in the vegetable matrix; however, above 80%, the material become too wet, causing water to flow away from the matrix. As illustrated in Table 1, as the hydration ratio increased, the yield in terms of extract volume also increased, reaching 43.5% with 80% hydration. 

Likewise, various parameters affect UAE extraction, such as solvent concentration, frequency, temperature, and sonication time, which must be optimized depending on matrix type, accordingly to the literature [33].

Due to polyphenols’ hydrophilicity rather than lipophilicity, hydroalcoholic solutions are widely used in polyphenol extraction [34]. Specifically, previous studies have used methanol–water mixtures to extract polyphenols from grapes and their by-products [35,36]. Also, in our study, the GP was UAE-extracted using MeOH/H_2_O (1:1, *v*/*v*) as an extracting solvent. In the UAE extraction, ultrasonic baths were operated in sweep-frequency modes at 40 kHz; frequencies between 20 and 60 kHz do not affect the stability of bioactive phenolic compounds [37].

Furthermore, studies in the literature have reported that temperatures of 20 to 50 °C increased the extraction yield without degrading phenolic compounds [5,38]. To prevent degradation processes, which may proceed more or less slowly at higher temperatures, the ultrasonic bath temperature was set at 20 °C. In addition, the ultrasound extraction cycle lasted 30 min, which was sufficient to obtain the maximum yield according to [38]. During the first phase of extraction, known as washing, 90% of the total phenolic compounds can be recovered. This phase lasts for 10 to 20 min. The second one takes 60 to 100 min and is called “slow extraction” [5,38].

### 3.2. Chemical Characterization

To fully understand the metabolic composition of this undervalued by-product, an array of techniques were used. In detail, ATR-FTIR and HPLC-UV-DAD techniques were applied to unravel the chemical composition.

#### 3.2.1. Spectroscopic Profile by ATR-FTIR

**Figure 2:**
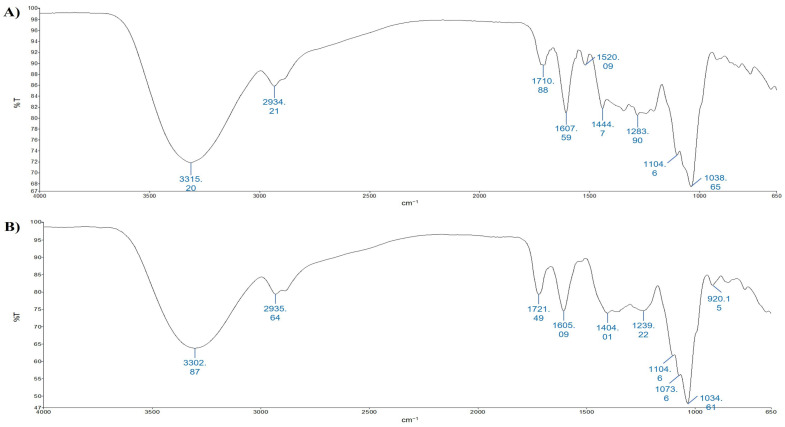
ATR-FTIR spectra of TOT-UAE (panel (**A**)) and TOT-MHG (panel (**B**)). Peaks are identified by numbers.

Firstly, TOT-UAE and TOT-MHG extracts from the Aglianico GP were qualitatively analyzed with ATR-FTIR (Figure 2A,B); the comparison was done based on peaks in the 4000–650 cm^−1^ spectral region [39]. Vibrational mode and peak assignments are based on those in the literature. Table 2 lists the wavelength of the peak absorbance.

The spectra consist of several bands resulting from the contribution of the vibrational mode of different functional groups. The broad band present in the extracts ranging from 3680 to 3000 cm^−1^ may be due to hydroxyl groups (-OH) and C–H stretching. It should be noted that phenolic compounds are distinguished by the presence of -OH, which resonates in this area. The spectral range 3000–2800 cm^−1^ shows a band at 2934 cm^−1^ for TOT-UAE and 2936 cm^−1^ for TOT-MHG, and a shoulder at 2885 cm^−1^ for both extracts due to methyl/methylene/methyne/methoxy/methyl ether C–H asymmetrical/symmetrical stretch. In the range 1720–1520 cm^−1^, the TOT-UAE extract shows three well-resolved peaks (1711, 1608, and 1520 cm^−1^; Figure 2A), while two peaks (1721 and 1605 cm^−1^; Figure 2B) are present in the TOT-MHG extract. The band at about 1710 cm^−1^ is due to carboxylic acid groups’ (C=O) stretching vibrations: according to the literature, the hydroxybenzoic and hydroxycinnamic acids show carboxylic acid groups (C=O) stretching vibrations in the 1715–1680 cm^−1^ region. The aromatic ring with six carbon atoms has links (C=C) that generate bands ranging around 1625 and 1430 cm^−1^; in our case, this could correspond to the band at 1608 cm^−1^ in the TOT-UAE extract (Figure 2A) and the band at 1605 cm^−1^ in the TOT-MHG extract (Figure 2B) [40]. Based on the literature, flavan-3-ols, including catechin, epicatechin and galloyl derivatives, contribute to the bands around 1240 cm^−1^ [41]. The range 1400–1200 cm^−1^ is not highly resolved in both extracts, while it is well resolved at 1104 cm^−1^; in this range, the spectrum of TOT-UAE allows for better discrimination between the bands (Figure 2A). The region 1170–945 cm^−1^ is characterized primarily by the carbohydrate functional groups. Particularly, the spectrum of TOT-UAE shows a maximum at 1039 and smaller bands at 1105 and 1075 cm^−1^, while the spectrum of TOT-MHG shows a maximum at 1035 cm^−1^ with shoulders at 1105 and 1074 cm^−1^. These bands are assigned to mono- and polysaccharides with the absorptions of stretching vibrations of C–O (mono-, oligo- and carbohydrates) [42].

To maximize the recovery of polyphenol compounds, the fractionation of the two parental extracts (TOT-UAE and TOT-MHG) was carried out: acidified water was used to recover sugars, organic acids, and salts, ethyl acetate was used to recover non-anthocyanin phenols, including flavonoids and flavan-3-ols, and acidified methanol was used for anthocyanins. F1 (UAE and MHG) corresponds to the sugar and polysaccharide components; they show a strong band with a maximum at 1027 cm^−1^ and a broad band centered to about 3300 cm^−1^ for both extracts, due to hydroxyl groups (-OH) and C–H stretching (Figure S1A,B). 

**Figure 3:**
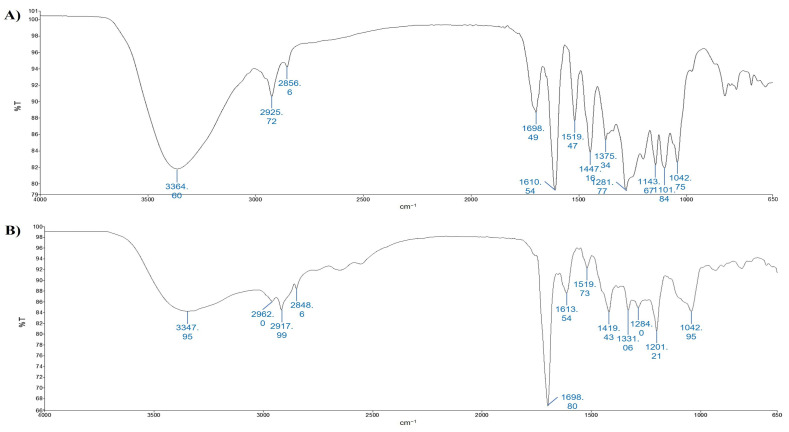
ATR-FTIR spectra of F2-UAE (panel (**A**)) and F2-MHG (panel (**B**)). Peaks are identified by numbers.

In Figure 3, the spectra of the non-anthocyanin phenolics fractions are shown. 

**Figure 4:**
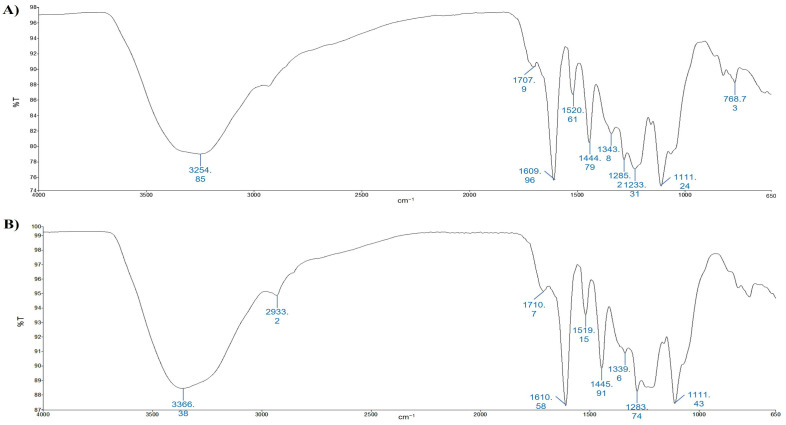
ATR-FTIR spectra of F3-UAE (panel (**A**)) and F3-MHG (panel (**B**)). Peaks are identified by numbers.

According to [43], characteristic vibrations of hydroxyl groups are observed in the wavenumber area of 3700–3100 cm^−1^, with a band due to absorption at a wavelength of 3348 cm^−1^ corresponding to the stretching of the OH groups (Figure 3A,B) [43]. The second strong peaks occur around 1698 and 1699 cm^−1^ for F2-UAE and F2-MHG, respectively, and are associated with carbonyl band stretching (C=O). The stretching of the C=C–C aromatic bond appears at 1611 and 1614 cm^−1^ for F2-UAE and F2-MHG, respectively. The presence of sugar in glycosylated phenols is responsible for the spectral region around 1047 cm^−1^. Spectra of anthocyanin fractions (F3-UAE and F3-MHG) are shown in Figure 4A,B. The major peak, in the range of 3700 to 2900 cm^−1^, corresponds to the OH stretch; it is very similar to that of pomace extracts. It is interesting to highlight the strong decrease in the band around 1700 cm^−1^ that is well visible in F2, and the presence of two bands at 1610 cm^−1^ (more evident in F3-MHG) and 1445 cm^−1^ (more important band in F3-UAE). The ATR-FTIR spectra of the two anthocyanin fractions appear very similar, even if with different proportions between bands.

#### 3.2.2. Metabolic Profile of Non-Anthocyanin Phenolics Fractions by HPLC-UV-DAD

**Figure 5:**
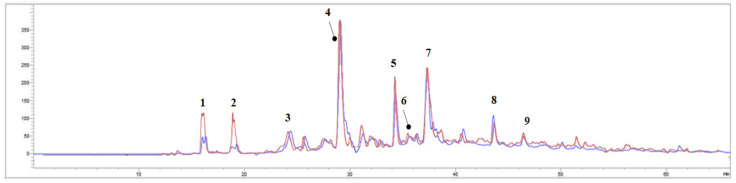
HPLC-UV chromatogram recorded at 280 nm of F2-UAE (blue line) and F2-MHG (red line). The compounds identified have been marked with a number.

An assessment of the major phenolic compounds present in the non-anthocyanin phenolics fractions (F2-UAE and F2-MHG) and anthocyanin phenolics fractions (F3-UAE and F3-MHG) was performed by HPLC-UV-DAD. The chromatograms registered at 280 nm are reported in Figure 5 (F2-UAE: blue line; F2-MHG: red line). All the constituents were tentatively identified considering the data provided in the literature and the spectral behaviours of reference standards, if available. The most abundant compound in both profiles is represented by catechin (peak **4**), the flavan-3-ol most present in grapes, as well as pomace [15,44]. It has previously been found in Aglianico seeds, which were more catechin-rich than their epicatechin isomer (peak **7**) [45]. Peak **5** has been identified as procyanidin B2, a proanthocyanidin found mainly in grape seeds [46,47]. The extractability of some compounds may be related to the process of making red wine versus white wine [46]. Peaks **1** and **2** have been characterized as gallic acid hexoside and gallic acid, respectively. Their relative abundances are different in the two extracts, with TOT-MHG exhibiting a greater amount. This phenolic acid, together with protocatechuic acid (peak **3**), is a common bioactive compound found in GP, particularly from red grapes [48,49]. Metabolites **8** and **9** were identified as gallocatechin gallate (GCG) and epicatechin-3-*O*-gallate (ECG), respectively. Furthermore, a study of the chromatogram recorded at 320 nm aided us in identifying compound **6** as 4-*O*-caffeoylquinic acid. 

#### 3.2.3. Study of the Anthocyanin Profile by HPLC-UV-DAD

**Figure 6:**
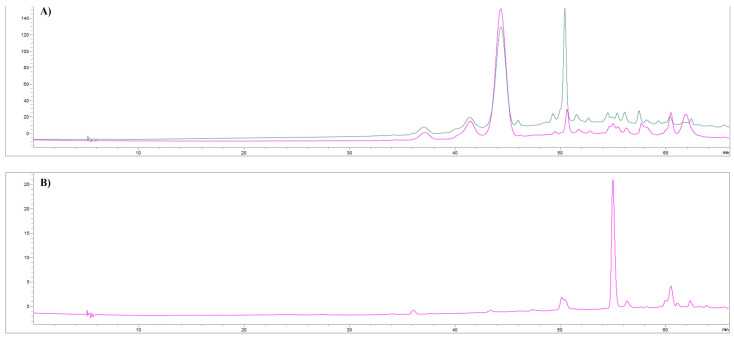
HPLC-UV chromatograms recorded at 520 nm of F3-UAE (magenta line) and F3-MHG (greyish cyan line) (panel (**A**)), and of anthocyanin fraction subjected to acid hydrolysis (panel (**B**)).

The F3 compounds (UAE and MHG) derived from SePak were analyzed by HPLC-UV-DAD. The chromatograms recorded at 520 nm (Figure 6A) show that F3-UAE (magenta line) and F3-MHG (greyish cyan line) consist of several anthocyanins and abound in two kinds of them, present at the retention times of 44.361 min (peak **1**) and 50.415 min (peak **2**), respectively. The relative abundances of the two peaks are different in F3-UAE and F3-MHG. In detail, in F3-UAE, the ratio of the heights of the two peaks is 0.84:0.16; on the other hand, in F3-MHG, it is 0.47:0.53. 

Peak **1** was identified as malvidin-3-*O*-glucoside chloride (oenin chloride). The UV spectrum showed a λ_max_ at 276 and 527 nm (Figure S2). It is estimated that it is the most representative anthocyanin in cultivated grapes [50]. The identification of peak **2** was made possible by the analysis of the hydrolyzed chromatogram; the HPLC-DAD profile showed two different aglycones at retention times of 55.003 and 60.510 (Figure 6B). The first one was identified as cyanidin chloride, and the second one as malvidin chloride. In detail, peak **2** was tentatively identified as cyanidin glucoside, esterified by caffeic acid in the C6″ position of the glucose moiety (Figure S3A). The presence of caffeic acid moiety was deduced from the study of the UV spectrum of the residue after hydrolysis, with a peak at the retention time of 50.415 (Figure S3B). Beyond the λ_max_ characteristics of anthocyanins, the UV spectrum showed λ_max_ values at 217 nm and 325 nm, with a shoulder at 295 nm characteristic of caffeic acid [51,52]. The identification of the anthocyanin moiety as cyanidin was confirmed by the UV spectra showing distinctive bands in the 450–560 nm region (λ_max_ at 529 nm) and the 240–280 nm region (λ_max_ at 273 nm) (Figure S3C). The presence of malvidin chloride as aglycone confirms the identification of peak **1** as malvidin-3-*O*-glucoside chloride.

### 3.3. Total Polyphenol Content 

**Figure 7:**
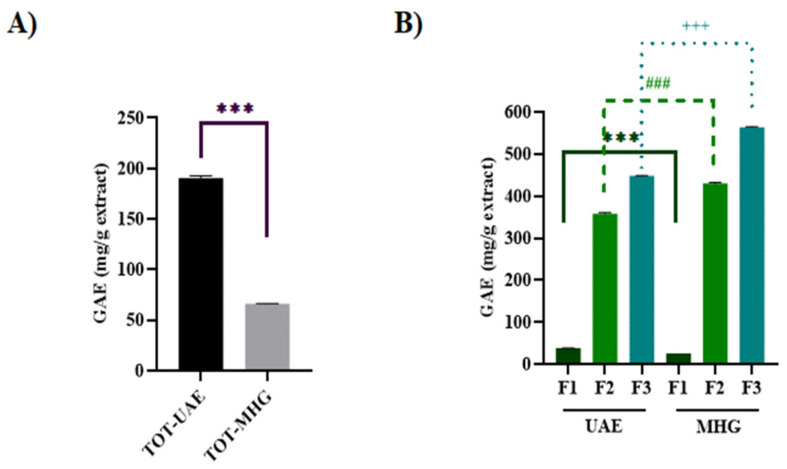
TPC of TOT-UAE and TOT-MHG (panel (**A**)), and of F1-, F2- and F3-UAE and F1-, F2- and F3-MHG (panel (**B**)). Results are expressed as mg of GAE per g of extract as reported in Material and Methods. Symbols indicate significance: *** *p* < 0.001 UAE F1 *vs*. MHG F1, ^###^ *p* < 0.001 UAE F2 *vs*. MHG F2, and ^+++^ *p* < 0.001 UAE F3 *vs*. MHG F3 (ANOVA test).

TPC varies greatly according to the different extraction methods and the solvent used [53]. The two extraction techniques employed in this work highlight significant differences in the phenolic contents of the two GP extracts, as reported in Figure 7. The highest TPC of 190.25 mg GAE/g was found in the TOT-UAE extract, while the lowest TPC (66.16 mg GAE/g) was noted in the TOT-MHG extract (Figure 7A). The different behaviours of the two parental extracts are probably due to the type of extraction performed: in UAE, the shock waves cause the mechanical breaking of the cell walls and, together with the different polarities and solubilities of the extractants, this favoured the preparation of the extracts [54]. On the contrary, MHG lacks an extraction solvent; so, using “*in situ*” water in the matrix, the hydrophilic phytoconstituents are extracted [55]. In detail, access to the matrices is made possible by microwave heat generation, leading to the damage and weakening of the matrix tissue [56]. Considering that the sugar content of GP is approximately 15 g per 100 g and given the affinity of these molecules for water, it is plausible that in the aqueous extract (TOT-MHG), the abundance of saccharide components was much higher than in the hydroalcoholic one (TOT-UAE) [57,58]. For this reason, the two parental extracts were fractionated through a C-18 cartridge to separate anthocyanin and non-anthocyanin phenolics from more polar compounds. The data acquired (Figure 7B) underline that F1-UAE and F1-MHG had the lowest TPCs (38.52 mg GAE/g and 26.39 mg GAE/g, respectively) due to their richness in polar compounds, particularly sugar. Moreover, F2-MHG and F3-MHG showed phenol contents 6.5 and 8.5 times higher than that of the parental extract (TOT-MHG, 66.16 mg GAE/g), respectively. In the same way, F2-UAE and F3-UAE showed phenol contents 1.88 and 2.36 times higher than that of the TOT-UAE (190.25 mg GAE/g). This suggests an effective removal of the saccharide backbone molecules (F1) and a higher concentration of the phenolic substances (F2 and F3).

Furthermore, we found that F2-MHG and F3-MHG had higher TPCs compared to F2-UAE and F3-UAE, which were statistically different (Figure 7B). However, the TPC of F1-UAE was greater than that of F1-MHG (Figure 7B), reflecting the differences in the chemical compositions yielded by the two extraction methods. 

### 3.4. Determination of Radical Scavenger Capacity 

**Figure 8:**
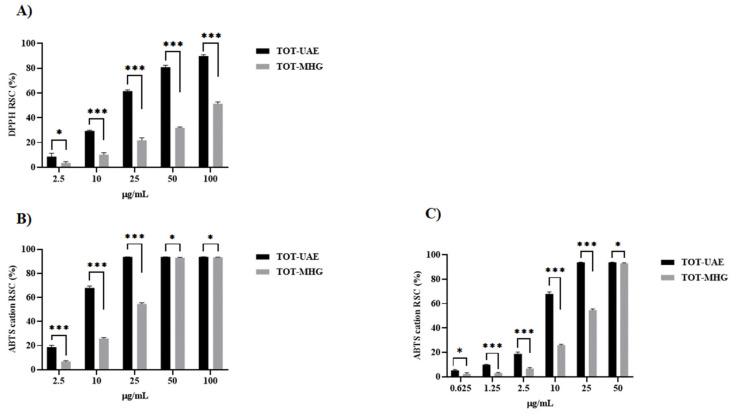
RSC (%) of TOT-UAE and TOT-MHG *vs*. DPPH radical (panel (**A**)) and ABTS radical cation (panels (**B**,**C**)). Symbols indicate significance: * *p* < 0.05, and *** *p* < 0.001 UAE *vs*. MHG (ANOVA test).

The *in vitro* radical scavenger capacity was evaluated through two different assays, DPPH^●^ and ABTS^●+^. According to its greater GAE content per gram of extract compared to TOT-MHG, TOT-UAE was found to be more reactive. In detail, it exerted a dose-dependent radical scavenging capacity measured by DPPH assay, with an ID_50_ value equal to 16.79 µg/mL (Figure 8A). Similarly, TOT-UAE showed an extremely high ability to scavenge ABTS^●+^, even at the tested dose of 25 µg/mL (Figure 8B). This implied that the sample should be further diluted down to 0.625 µg/mL. An ID_50_ value of 5.27 µg/mL was calculated (Figure 8C). On the other hand, the TOT-MHG extract showed a milder efficacy in relation to the two probes used compared to TOT-UAE. In detail, the estimated ID_50_ values were 93.00 µg/mL (Figure 8A) and 25.16 µg/mL (Figure 8C).

**Figure 9:**
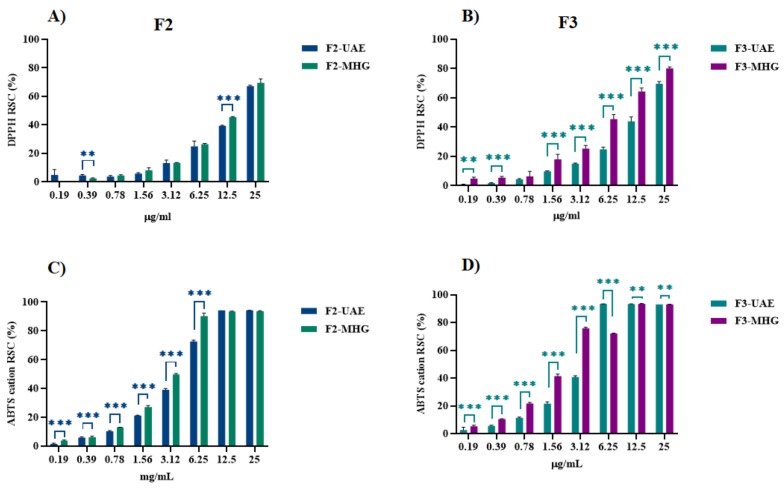
RSC (%) of F2 and F3 produced by UAE and MHG *vs*. DPPH radical (panels (**A**,**B**)) and ABTS radical cation (panels (**C**,**D**)). Symbols indicate significance: ** *p* < 0.01, and *** *p* < 0.001 UAE *vs*. MHG (ANOVA test).

Also, the fractions obtained from SPE were assessed for their ability to scavenge DPPH^●^ and ABTS^●+^. The derived F1-UAE and F1-MHG proved to be ineffective in relation to both the DPPH radical and the ABTS radical cation. The acquired data, shown in Figure 9A–D, show an increase in the activity of the organic fractions (F2 and F3). In this regard, F3-MHG showed a marked scavenging activity with both DPPH^●^ and ABTS^●+^, with relative ID_50_ values equal to 7.04 µg/mL and 1.80 µg/mL. On the other hand, F2-UAE and F3-UAE, while presenting good reducing efficacy, showed lower ID_50_ values with respect to the same fractions derived from the fractionation of the MHG extract. 

### 3.5. Anti-Proliferative Effects of F3-UAE and MHG on HT-29 Cell Line

**Figure 10:**
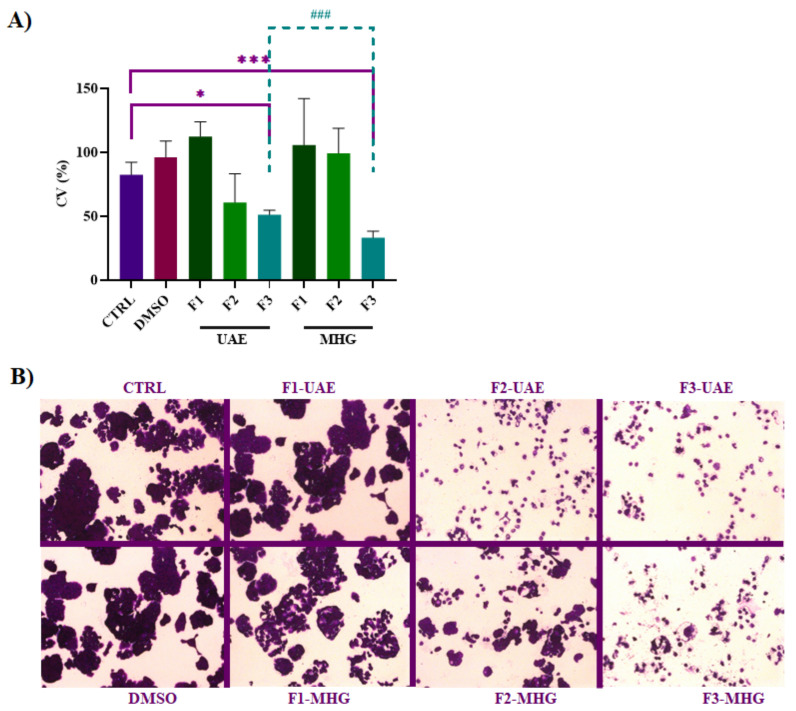
Effects of F1, F2, and F3 from UAE and MHG on HT-29 cell viability. (**A**) Cells were treated for 72 h with F1, F2, and F3 (100 µg/mL) and a crystal violet assay was performed as reported in Materials and Methods. Results are expressed as % of cell viability (CV). Data are expressed as mean ± SD. Symbols indicate significance: * *p* < 0.05, and *** *p* < 0.001 with respect to CTRL (DMSO) and ^###^ *p* < 0.001 F3-MHG *vs*. F3-UAE (ANOVA test). (**B**) Photographs of cells incubated with F1, F2, and F3 were taken on an optical microscope Axiovert 200 M Zeiss; 200, BF.

To evaluate their abilities to reduce cell proliferation, F1, F2, and F3, as yielded by UAE and MHG, have been tested on the HT-29 cell line. Cell proliferation was slightly increased following F1-UAE and F1-MHG treatments compared to CTRL cells (DMSO) (Figure 10A,B). Based on the chemical composition of F1, which consists mainly of sugar residues, this result matches the assessment that the glucose transporter GLUT1 in colorectal adenocarcinoma cell lines is involved in cell proliferation [59]. As reported in Figure 10A, F2-UAE and F2-MHG did not significantly reduce cell viability with respect to CTRL. Flavan-3-ols are among the most abundant compounds in both F2-UAE and F2-MHG, as shown in Figure 5. These compounds do not appear to be able to significantly modulate cell proliferation at the tested concentrations. It is noteworthy that several factors influence the bioactive effects of polyphenols on cancer cells. The first of these is the concept of polyphenol accessibility, which is influenced by the relative amount of compounds released, as well as the efficiency of polyphenol passage across the intestinal epithelium [18]. Instead, the numbers of cells were significantly reduced by 30% and 50%, respectively, after treatment with F3-UAE and MHG (100 μg/mL). F3-MHG had a significantly greater effect than F3-UAE, likely due to the presence of different specific compounds according to HPLC analysis (Figure 6). Specifically, we assume that the different ratios of the two peaks, identified as malvidin-3-*O*-glucoside chloride and cyanidin 3-(6-*p*-caffeoyl)glucoside, could support the different biological effects, which need to be investigated further. Accordingly, the data in the literature suggest the effects of anthocyanin-rich extracts in inhibiting the growth of human colon adenocarcinoma cells by the modulation of the apoptosis process [60]. 

**Figure 11:**
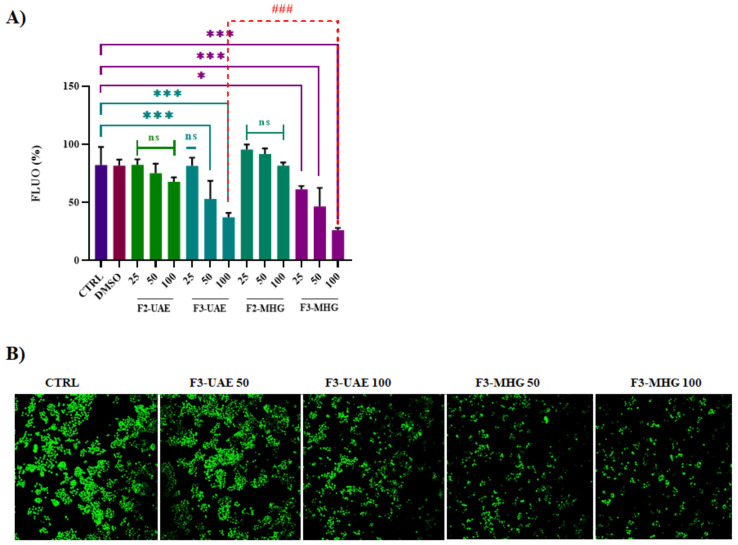
Antiproliferative effects of F3-UAE and F3-MHG on HT-29 cell viability. (**A**) After 72 h of treatment with F2-UAE, F2-MHG, F3-UAE and F3-MHG (100 µg/mL), the CyQuant assay was performed as reported in Materials and Methods. Values are presented as mean ± SD. Symbols indicate significance: * *p* < 0.05, and *** *p* < 0.001 with respect to CTRL (DMSO) and ^###^ *p* < 0.001 F3-UAE *vs*. F3-MHG (100 µg/mL) (ANOVA test). (**B**) Photographs of cells incubated with 50 and 100 µg/mL of F3-UAE and F3-MHG were taken on an optical microscope Axiovert 200 M Zeiss; 200, FITC.

The effect on cell viability of non-anthocyanin and anthocyanin fractions (F2-UAE, F2-MHG, F3-UAE and F3-MHG) was also assessed by CyQuant assay by testing different concentrations (25, 50 and 100 μg/mL, *w*/*v*), as reported in Figure 11A,B. The numbers of cells were significantly reduced by about 50 and 60% for F3-UAE and F3-MHG at the higher concentration (100 μg/mL), and by about 30 and 40% at 50 μg/mL, respectively. F3-MHG was also found to be more effective than F3-UAE (100 μg/mL), confirming the better efficacy of F3-MHG in reducing the proliferation of HT-29 cells.

## 4. Conclusions

The agricultural and food industries produce many non-edible wastes and by-products that can be repurposed due to their richness in active phytochemicals. In the context of a circular economy, this approach reduces raw material, environmental and energy waste. Therefore, sustainable and green methods must be used to extract value-added compounds from this waste. As a result of their advantages over conventional extraction techniques, novel extraction methods have also attracted the attention of researchers. One of these is MHG, an application that expands MAE’s range further and makes it a more environmentally friendly process. Nevertheless, comparing the biological activities of extracts obtained by novel and green extraction techniques such as MHG with those obtained by traditional extraction methods poses a significant challenge. Here, MHG and UAE were performed to obtain a polyphenol-enriched extract from GP. MHG has been proven to be an effective strategy for recovering polyphenol compounds, and the fractionation by SPE was ascertained through ATR-FTIR and HPLC analysis, with improved radical scavenger performance when compared with UAE fractions. In addition, the anthocyanin fraction enriched by MHG reduced HT-29 cell growth by 70%, suggesting the need for further research. Ultimately, MHG is an effective method to produce polyphenol-enriched fractions with interesting biological properties. 

## Figures and Tables

**Table 1:** Extracts yield (mL) *vs*. water percentage used for rehydration of GP.

% Hydration	Yield (mL)
25	20
50	74
60	99
70	159
80	174

**Table 2:** Vibrational mode of the spectra of TOT-UAE and TOT-MHG extracts in light of recent literature. The range values of the peaks are reported.

Wavenumber (cm^−1^)				Assignments
TOT-UAE		TOT-MHG		
Position	Intensity	Position	Intensity	
3315	0.1435	3302	0.1952	OH and C–H stretching
2997	0.0522	2998	0.0737	CH_2_ and CH_3_ stretching vibrations
2934	0.0662	2933	0.1001	
2167	0.0088	2167	0.0145	
1711	0.0471	1721	0.1	Carbonyl C=O stretching
1668	0.0321	1666	0.0583	Aromatic ring C=C stretching
1608	0.0915	1605	0.1274	
1551	0.0339	1509	0.047	C–O stretching vibrations
1520	0.0472	1404	0.1313	C–H bending
1501	0.0375	1300	0.1162	O–H bending
1284	0.094	1239	0.1269	C–N stretching
1172	0.0646	1172	0.0868	Aromatic C–H in plane bend; C–O stretching vibrations
1038	0.1711	1035	0.3191	
944	0.0358	945	0.0708	
		920	0.0861	C–H deformation vibrations, out-of-plane bend
		881	0.072	

## Data Availability

The data used to support the findings of this study can be made available by the corresponding author upon request.

## Supplementary Materials

The following supporting information can be downloaded at: https://www.mdpi.com/article/10.3390/foods12142678/s1, Figure S1: ATR-FTIR spectra of F1-UAE (panel **A**) and F1-MHG (panel **B**). Peaks are identified by numbers; Figure S2: UV-DAD spectrum of malvidin-3-*O*-glucoside chloride identified in F3 (UAE and MHG); Figure S3: UV-DAD spectrum of cyanidin 3-(6-*p*-caffeoyl)glucoside identified in F3 fractions (panel **A**); UV-DAD spectrum of caffeic acid moiety of the residue after acid hydrolysis (panel **B**); UV-DAD spectrum of cyanidin identified in F3 fractions as aglycon after acid hydrolysis (panel **C**).

## Abbreviations

ABTS	2,2′-azinobis-(3-ethylbenzothiazolin-6-sulfonic acid)
ATR-FTIR	Attenuated total reflectance–Fourier transform infrared spectroscopy
BF	Bright field
CV	Cell viability
DMEM	Dulbecco’s modified Eagle’s medium
DMSO	Dimethyl sulfoxide
DOCG	Controlled and guaranteed denomination of origin
DPPH	2,2-diphenyl-1-picrylhydrazyl
DTGS	Deuterated tri-glycine sulfate
ECG	Epicatechin-3-*O*-gallate
FBS	Fetal bovine serum
GAE	Gallic acid equivalents
GCG	Gallocatechin gallate
GP	Grape pomace
HPLC-UV-DAD	High-performance liquid chromatography–ultraviolet–diode array detection
MHG	Microwave hydrodiffusion and gravity
PBS	Phosphate-Buffered Saline
RSC	Radical scavenging activity
SD	Standard deviation
SFME	Solvent-Free Microwave Extraction
SPE	Solid-phase extraction
TPC	Total phenol content
UAE	Ultrasound-assisted extraction
