# Supplementary material for: A Comparative Study between Microwave Hydrodiffusion and Gravity (MHG) and Ultrasound-Assisted Extraction (UAE): Chemical and Biological Characterization of Polyphenol-Enriched Extracts from Aglianico Grape Pomace
Source: Foods. 2023 Jul 11;12(14):2678. doi: 10.3390/foods12142678 (PMC10378583; doi:10.3390/foods12142678)
Supplement: Supplementary file 1 [file foods-12-02678-s001.zip › foods-2443828-supplementary.pdf]

Supplementary materials

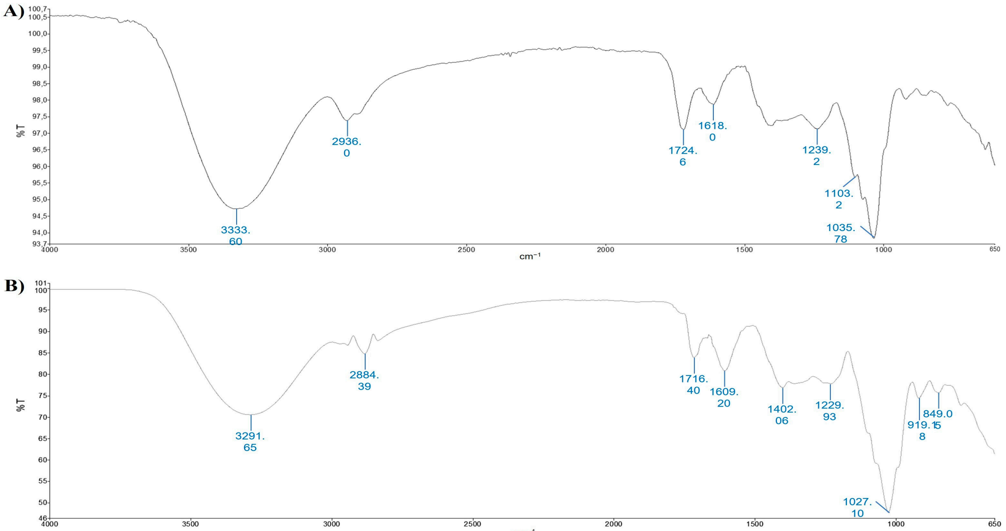

Figure S1. ATR-FTIR spectra of F1-UAE (panel **A**) and F1-MHG (panel **B**). Peaks are identified by numbers.

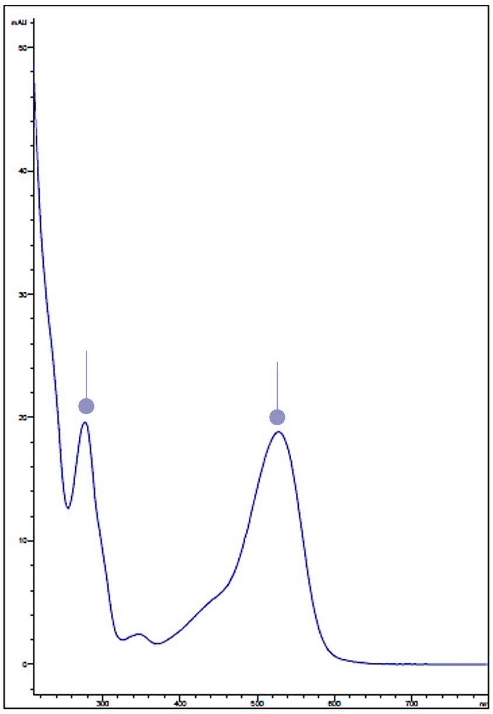

Figure S2. UV-DAD spectrum of malvidin-3-*O*-glucoside chloride identified in F3 (UAE and MHG).

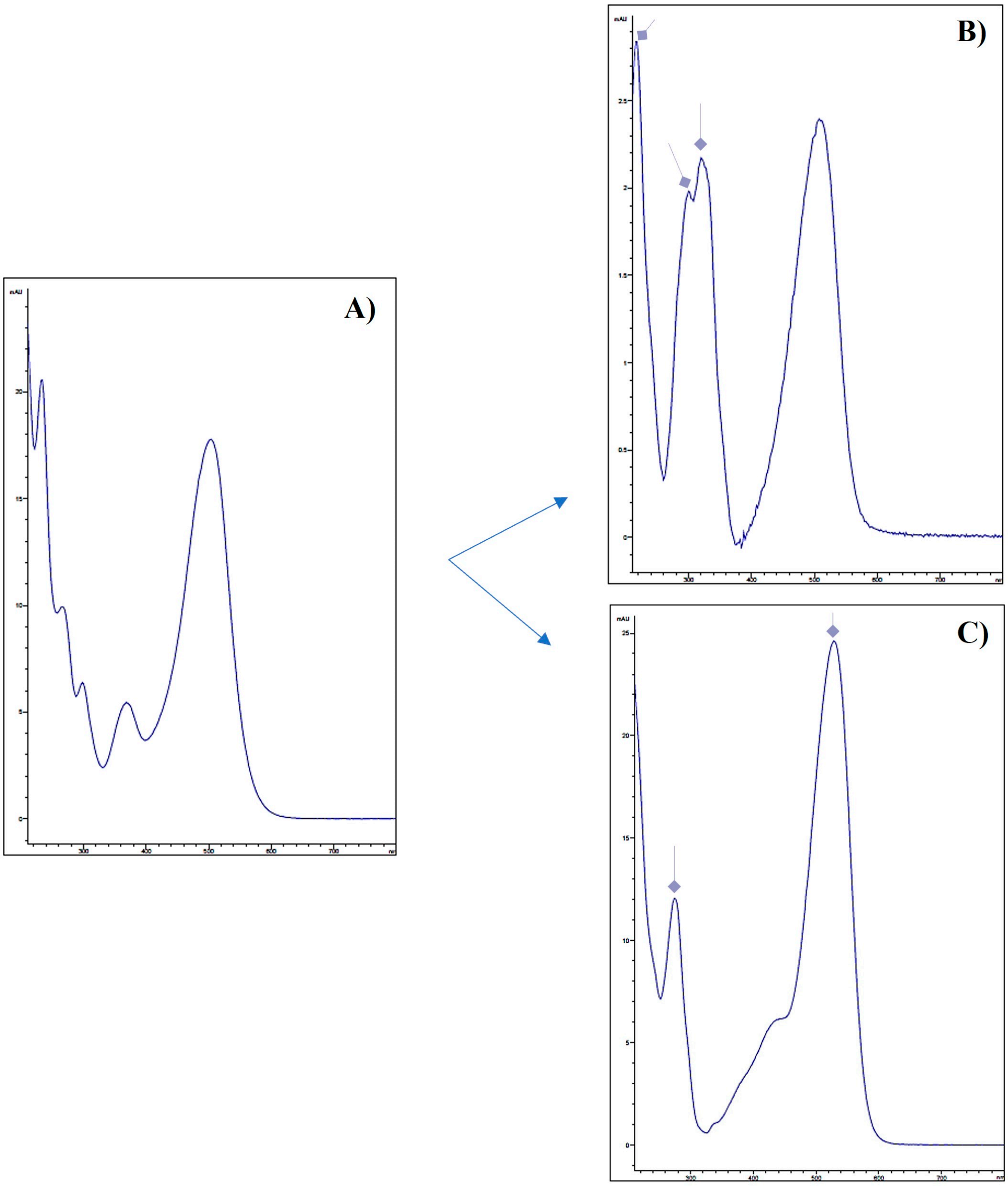

Figure S3. UV-DAD spectrum of cyanidin 3-(6-*p*-caffeoyl)glucoside identified in F3 fractions (panel **A**); UV-DAD spectrum of caffeic acid moiety of the residue after acid hydrolysis (panel **B**); UV-DAD spectrum of cyanidin identified in F3 fractions as aglycon after acid hydrolysis (panel **C**).
